# Barriers and Facilitators to Community Mobility for Assistive Technology Users

**DOI:** 10.1155/2012/454195

**Published:** 2012-09-13

**Authors:** Natasha Layton

**Affiliations:** School of Health and Social Development, Deakin University, 221 Burwood Highway, Burwood, VIC 3125, Australia

## Abstract

Mobility is frequently described in terms of individual body function and structures however contemporary views of disability also recognise the role of environment in creating disability. *Aim*. To identify consumer perspectives regarding barriers and facilitators to optimal mobility for a heterogeneous population of impaired Victorians who use assistive technology in their daily lives. *Method*. An accessible survey investigated the impact of supports or facilitators upon actual and desired life outcomes and health-related quality of life, from 100 AT users in Victoria, Australia. This paper reports upon data pertaining to community mobility. *Results*. A range of barriers and enablers to community mobility were identified including access to AT devices, environmental interventions, public transport, and inclusive community environs. Substantial levels of unmet need result in limited personal mobility and community participation. Outcomes fall short of many principles enshrined in current policy and human rights frameworks. *Conclusion*. AT devices as well as accessible and inclusive home and community environs are essential to maximizing mobility for many. Given the impact of the environment upon the capacity of individuals to realise community mobility, this raises the question as to whether rehabilitation practitioners, as well as prescribing AT devices, should work to build accessible communities via systemic advocacy.

## 1. Introduction

Getting around at the home and in the community is a core activity, central to much human participation and therefore of key interest to rehabilitation practitioners. Identifying the constraints and supports which consumers perceive as impacting their current and desired life outcomes will both inform the work of rehabilitation practitioners and identify any barriers usually beyond the gaze of rehabilitation practice [1]. 

### 1.1. Mobility

Mobility, defined by the Oxford Dictionary of English [2] as the capacity to move, is a core element of human capacity. Independent mobility, preferably without the need for assistive technology (AT), is viewed as a key outcome measure, alongside communication and self-care, in the rehabilitation literature [3]. Health-related quality-of-life measures also regard the capacity to independently mobilize as a key indicator for quality of life [4, 5].

The extent of mobility will depend upon both the capacity of the person and the nature of the environments in which the person operates. A tension exists in considering the relationship between the person and the environment in which mobility takes place. The rehabilitation approach typically addresses the capacities and deficits within the individual and therefore predominantly locates the mobility issue with the person [6]. This is at odds with social models of disability where barriers present in the environment are seen as disabling, and the dismantling of these barriers will minimize disablement [7, 8]. Recent disability theorists acknowledge the influence of both individual impairment effects and barriers within the environment which may need addressing [9–11]. To some extent such contemporary conceptualizations of disability are realized in the biopsychosocial model put forward by the International Classification of Functioning, Disability and Health (ICF) [12]. Mobility is classified as one complete ICF activity and participation chapter (chapter 4 page 138) and underpins performance in a number of other chapters, for example, Other Life Areas (chapter 8 page 164) and Community, Social & Civic Life (chapter 9: 168). A third section of the ICF framework is that of Environmental Factors, which “make up the physical, social and attitudinal environment in which people live and conduct their lives” (page 171) [12]. In line with the social model of disability then, the ICF acknowledges barriers and facilitators which may impact human performance, beyond body structures and functions [13]. 

### 1.2. Mobility within Health and Human Rights Frameworks

In Australia as with other countries, the importance of mobility is enshrined in policy goals at various levels of government [14, 15]. Australia is a signatory to the Convention on the Rights of Persons with Disabilities (CRPD) [16] and mobility is identified as a key right within this. “General principles” (Article 3) describes “full and effective participation and inclusion in society” and “equality of opportunity and accessibility”, while Article 19 addresses “Living independently and being included in the community” and Article 20 “Personal mobility”. “Accessibility” (Article 9) directs signatories to

*“take appropriate measures to ensure to persons with disabilities access, on an equal basis with others, to the physical environment, to transportation, to information and communications, including information and communications technologies and systems, and to other facilities and services open or provided to the public, both in urban and in rural areas” [16].*

These frameworks open the way for a more multilayered response to the complexities involved in mobilizing, including the nature of the environment(s) in which mobilization occurs [13]. 

### 1.3. What Interventions Enable Mobility? Enablers and AT Solutions

AT devices, environmental interventions (EI), and personal care or support are key interventions which can be used throughout the lifespan, across occupational roles and at various stages of the disease trajectory [17]. Most effective when used in combination, they represent the key means by which people living with disabilities maximize their capacity for participation [18]. These interventions and supports have been termed “enablers”, in that they enable performance in life domains of importance [19]. While these enablers have generally been researched separately, relationships between them are emerging, for example, the impact of assistive technology upon personal care use [20, 21] and the relationship of assistive technologies and environmental interventions [22, 23]. Evidence for the interrelationship of these three enablers is found in the Equipping Inclusion Studies [24], from which data for this paper is drawn. Here, two-thirds of participants utilized an individualised combination of all three enablers in their daily lives [24]. This study provides empirical evidence to support the notion of the Assistive Technology Solution (AT solution), defined as follows:

*“as an individually tailored combination of hard (actual devices) and soft (assessment, trial and other human factors) assistive technologies, environmental interventions and paid and/or unpaid care” [25].*

The concept of an “AT solution” is useful in describing the suites of facilitators or enablers used to engage in community mobility elicited in the study described below.

## 2. Method

Victoria's Aids and Equipment Action Alliance (AEAA) [26] is a nonprofit, multimember group consisting of people with disabilities, advocates, health professionals, and service providers. In 2008 the AEAA commissioned *The Equipping Inclusion Studies* [24], a series of mixed methods studies intended to investigate the impact of AT solutions upon the lives of adults with a disability. Deakin University ethics approval was gained to sample 100 users of assistive technology devices, from the population of those 18 years and over with disabilities in Victoria, Australia. In line with participatory research principles, AEAA provided a stakeholder reference group to advise the researchers particularly in the areas of recruitment, data analysis, and dissemination.  Table 1 outlines the research questions and methods pertaining to The Equipment Survey which is one aspect of *The Equipping Inclusion Studies* and is the source of the data presented in this paper. 

### 2.1. Survey Tool and Pilot

A 60-question survey was devised to identify the range of AT devices and other enablers (the elements of each persons' AT solution) used by participants. Open-ended responses describing the nature and extent of activities and participation enabled by AT use were prompted for a range of life areas [27]. Additionally, information was sought regarding potential improvements envisioned by participants and the impact of current and potential AT solutions upon difficulty levels and time use. These questions formed the first section of the survey, with a health-related quality-of-life measure (AQoL 6D) [5] forming the second section, and demographic questions as a final section. The survey was piloted with a sample of 9 individuals with disabilities and underwent minor edits prior to distribution. A key methodological challenge with the extensive question-set related to survey completion. The pilot process validated the decision to allow questions to be left incomplete, in order to enable participants to complete sections of meaning to them, despite the potential disadvantage of missing data. Responses were therefore included in analysis if they contained one or more responses in section one. 

### 2.2. Sample and Recruitment

The survey intended to capture a diverse sample in order to elicit a wide range of AT user experience. Invitations to participate in the survey were issued through the AEAA networks and through a range of Victorian health, community, and disability publications and alerts. Paper copies of the survey were also made available at disability support services and community health centres. To participate, participants sent in completed surveys, contacted the researcher to obtain a copy of the paper survey, or went to the advertised web link to fill in the accessible online survey. This could be completed and saved in stages due to its length. In order to elicit the responses of AT users themselves, as opposed to carers who may have additional or conflicting perspectives upon valued outcomes, proxy reporting was not used. Strategies to include AT users with a wide range of abilities included the offer of gift vouchers to the value of $20 in recognition for time spent in survey completion and the availability of personal assistance with scribing to complete the survey. The survey was available in a paper version as well as a fully accessible online survey format (see [28] for a comparative effectiveness report on accessible online surveys and description of this bespoke online survey tool devised for this study).

The survey produced quantitative and qualitative data, which was analysed using WHO International Classification of Functioning, Disability and Health [12], ISO 9999 Assistive Products for People with Disabilities [29], and UN Convention on the Rights of Persons with Disabilities [16].

## 3. Results

A wide cross-section of people with a disability responded to advertisements and snowball sampling methods via health and disability organizations, and word of mouth within the disability community. The decision to allow partial completion of the survey resulted in gaps including the demographic data; hence the data below identifies the proportion of participants who provided information on the demographic indices. 

### 3.1. Study Population

Eighty of the 100 respondents provided demographic information, identifying nearly 60 separate diagnoses, including 60% with physical disabilities, 14% with multiple conditions, and 13% with sensory disabilities. The remaining 13% did not complete this question. Most common conditions were neurological (stroke, polio, cerebral palsy, and spinal cord injury) followed by musculoskeletal (amputation, arthritic conditions). Fifty-nine percent were female and 41% male. Most were aged 45–64 years (39%) or 25–44 years (20%), with 13% over the age of 65 (28% did not complete this question). Sixty-two percent lived independently either alone or with a partner, 14% lived in the family home, and 2% resided in a specialist residential care facility (22% did not complete this question). Seventy-eight responded to questions regarding employment, and of these, 25% were employed and 20% identified substantial volunteering roles. Annual incomes were low, with 75% of participants receiving government pensions or allowances, 19% receiving part-time wages, and 6% in receipt of other income (not defined). Sixty-seven participants completed the health-related quality-of-life (HRQOL) measure. Analysis of this data showed the HRQOL of the study population to be less than half that of the norm for the Australian population (0.32 compared to 0.80) as measured by the Assessment of Quality of Life Measure (AQoL-6D) [30].

A substantial dataset emerged from the survey related to community mobility. These findings have been reanalysed here with a particular focus on the interface between individuals, their AT solutions, and the wider community in which mobility occurs. 

### 3.2. Participation in Life Areas Enabled by AT Solutions

Participants reported high utilization of three elements of an AT solution: devices; environmental interventions (including home modifications and the community environs), and personal care. Participants currently utilize an average of 13 items or elements within their AT solution (AT devices, environmental modifications, and personal care), averaging 9 AT devices each. In most cases (66%), all three elements were used by participants. Only 2% of participants relied on AT devices alone, while 16% used AT devices and personal care in combination and 15% used AT devices and environmental interventions together. 

**Figure 1:**
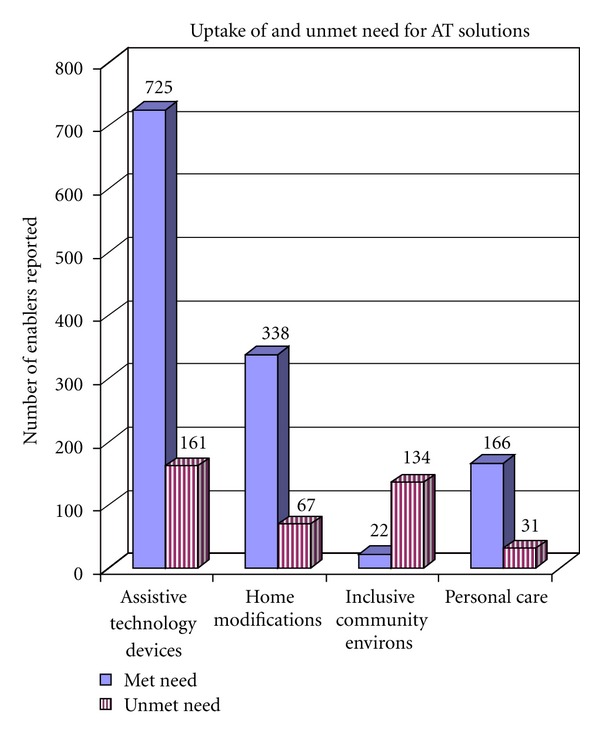
Uptake of and unmet need for AT solutions.

All 100 participants utilized individualised AT solutions to participate in multiple life areas, describing over 900 instances of engagement across the ICF activity and participation chapters [12].  Figure 1 contains overall-usage figures for AT devices (including mobility devices) and personal care.  Figure 1 also contains a breakdown of environmental interventions into home modifications and inclusive community environs—a theme which will be explored further in this paper and reflects enablers beyond the garden gate. 

Also shown in Figure 1 are total reported items of unmet need. As can be seen from the ratios pictured, provision was a major issue with significant undermet and unmet need reported by the study population. The figures shown in Figure 1 translate into unmet need of 6.5% for home modification, in comparison to 85.9% unmet need for inclusive community environs, which in part reflects the focus of government funding programs as discussed below. A critique of the Victorian government equipment funding scheme can be found in the full publication of *The Equipping Inclusion Studies *[24]. Of relevance to this paper was evidence of the lack of coverage of environmental interventions, despite their central importance to the effectiveness of other enablers and to the survey participants overall. As well as funding shortfalls and lack of response to life changes in the need for alterations to the home, the data evidenced substantial barriers in the wider community. As shown in Figure 1, these themes are reported separately as either home modifications or inclusive community environs. 

### 3.3. Facilitators and Barriers to Community Mobility: Interrelated Enablers

Elements of AT solutions were repeatedly seen to be effective in more than one area of activity. A number of respondents described making tradeoffs between activities. This involved rationing their participation based on insufficient enablers. Participants identified interdependent and overlapping enablers, describing situations where barriers in one area led to a need for more supports or enablers in other contexts leading to a decreased need for other supports.

*“If the environment was more accessible I wouldn't need any carer help. I do not use any now but sometimes it is difficult and I rely on friends to drag me up steps etc.”*

*“[I need] street changes—I use a chin-controlled chair and when I try to move the chair along street paths and cross the road, poorly constructed bumpy and steep crossovers are extremely difficult to navigate with my chin. When paths are not flat and smooth, my head moves too much for my chin to remain on the chin control, it makes it nearly impossible for me to get out in most areas locally like to the park or shop. The use of blue stones for crossovers is appalling for wheelchair users. [I need] better access into some buildings, venues and shops that haven't provided access for the disabled in wheelchairs”.*

### 3.4. Facilitators and Barriers to Community Mobility: The Experience of Constructing Individualised AT Solutions

Most respondents identified current difficulty levels of “moderate” to “moderate to severe” (3-4 on a 6-point scale) across life areas, and qualitative data showed dissatisfaction and frustration with current participation levels. Many participants described constructing an AT solution that worked for them as an ongoing process as their impairments, their life stages, and their occupational roles changed over time. Over 90% of participants identified ways in which their enablers could or should change in future, related either to ageing equipment, their own changing needs, or the desire for more effective participation:

*“I work full time, drive my own modified car. As I age, getting in and out of the car is getting more difficult. I may need further modification to the car to make it easier to get into… (I need) provision of an electric wheelchair so that I can go out as it is getting harder with age to wheel myself distances”.*

Frequently, participants felt unable to action needed changes due to a lack of resources and lack of responsiveness in the government equipment funder or lack of control over the external environment. For example, a maximum of $4,400 is allocated for home modifications once per lifetime, by the state government equipment funder. This covers less than 25% of the cost of a standard modification (e.g., modifying one entrance and one bathroom), and was reported to cause significant stress and difficulty in planning around life transitions or as physical needs alter,

* “As I get older my needs change. As my house is heritage listed it is difficult to change things inside, I would love to have wider doors, some rooms I cannot enter and the others I have a 2 cm leeway”.*

Another issue concerns the fact that the enablers identified were often mainstream products and services and therefore unfunded by government equipment funding schemes,

*“As I mainly use public transport I spend time planning on internet the route and means of transport (timetables) and how they connect. Often 1 Bus and 2 trains or 2 buses and 1 train to get somewhere; can be limited by access and the time of day/night travelling. (I use) a hand held GPS for navigating my way around the streets and electronic diary for planning, organising and remembering; telephone and mobile phone; computer with accessible technology [such as] screen reader”.*

In the above example, this participant with a degenerative condition is enabled by current mainstream technologies (GPS, internet access) which would require self-funding as they are not disability specific items available under the government equipment funding scheme. He is, however, limited by community enablers (timetables and public transport availability).

### 3.5. Classification and Provision of Enablers: AT Devices

The International Standards Organisation (ISO) provides an internationally accepted classification system for assistive products for persons with disability [29]. Products of specific relevance to mobility and access are covered in Chapter 12 (assistive products for personal mobility), and Chapter 18 (furnishings and adaptations to homes and other premises) [29]. Study participants used 243 mobility devices and identified unmet need for 55 mobility devices (see Table 2). Significantly, 18.6% of the AT device categories listed in the ISO are present on the State funding equipment list. The implications of this more than 80% shortfall is that, where users cannot self-fund, prescribers are substantially limited in the AT devices they can provide [31].

### 3.6. Classification of and Provision of Enablers: Environments

While ISO 9999 includes a section covering many environmental modification products in Chapter 18 Furnishings and adaptations to homes and other premises [29]; the WHO ICF was found to offer the most useful taxonomies for classifying environmental factors, in that all enablers fitted within either Chapter 1: Products and Technology; Chapter 2: Natural environment and human-made changes to environment; Chapter 3: Support and Relationships; Chapter 4: Attitudes; or Chapter 5: Services, systems and policies [12].

#### 3.6.1. Environment: Home Modifications

Ninety-six participants reported on home modifications. Modifications to the home were utilised by 39 of these respondents (43%) who described having a total of 332 home modifications. Forty-three respondents (45%) named 64 instances of unmet need for home modifications. While many home adaptations were desired as a result of changing circumstances such as ageing or a change in lifestyle and health, some were required as a result of poor architectural planning and regulation. 

#### 3.6.2. Classification of Enablers beyond the Garden Gate: Inclusive Community Environs

A diversity of factors in the community were described which related to, but moved beyond, the built environment alone. This emergent theme was termed “inclusive community environs” and sits alongside environmental interventions as a subset of the environment component of an AT solution (see Table 3). Twenty percent of participants explicitly named enabling community elements while over half of all participants (52%) identified barriers. This was the only category in which the level of unmet need exceeded current instances of provision. In total, 134 barriers were described within the environment beyond the garden gate. Of these, 48 concerned the need for universal design and physical access to environs and buildings (36% of unmet requirements), while 31 barriers related to lack of accessible public transport (23%) and 23 related to inaccessible public space (24%). Frequently, a lack of seamless infrastructure meant some accessibility initiatives did not translate into a realistic solution.

### 3.7. Compliance with the UN Convention on the Rights of Persons with Disabilities

The CRPD [16] framework was used during analysis to code the qualitative examples of activities and participations described by participants. Data was coded as instances of failing to realise CRPD principles in two ways. Firstly, the activity or participation event was recorded if it was experienced as difficult to the extent that participants were subjected to undue effort to participate or relinquished the task altogether. For example, an adult with a spinal cord injury described her difficulties with banking due to access, as follows:

*“Effort in running around finding accessible banks or embarrassing myself by yelling from the front door and having to be a dependent disabled person, reliant on people's good will.”*

Secondly, data was included in the CRPD analysis if the activities being described mapped directly to human rights expectations as expressed in the CRPD. The article with the highest level of noncompliance related to lack of AT provision was that of Accessibility (Article 9) with 32 instances of failure to realise reported; for example,

* “My rented flat has steps to get in the main entrance, so have to drive into downstairs car park, or come in the car entrance on the wheelchair. Ensuring all new apartment blocks with lifts have an entrance with no steps would be a big bonus! Also the apartment has a huge (20 cm) lip to get onto the balcony, so need to build a ramp.”*

Overall, 34 instances of participation consistent with the CRPD were explicitly named in the study data; however 134 instances of unsuccessful attainment relating to articles from the CRPD were also reported. Thus, the ratio of achievement to nonachievement against CRPD articles for the 100 participants in this study was approximately 1 : 4. This indicates that Australia has a substantial way to go in ensuring its obligations are met under the Convention. 

## 4. Discussion

The right to engage in a full life despite the presence of impairment is enshrined within human rights documents such as CRPD [16] and operationalised within health and disability frameworks such as WHO ICF [12]. Survey participants represented a small but varied cohort of adults with disability and provided evidence of the efficacy of AT devices, environmental interventions, and personal care. The voices of these participants demonstrated the capacity of many “user experts” to name barriers, identify plausible facilitators, and identify likely impacts and outcomes. These impacts and outcomes align well with broad conceptualizations of life such as those provided by ICF Activities and Participation Chapters [12]. Mobility is not seen as an end in itself, rather a necessary capacity in order to participate in occupations of meaning, as the following statement by a retired physicist living with postpolio syndrome illustrates:

*“Now that I need to use the manual wheelchair all the time, I cannot get it under the kitchen bench to make a cup of coffee as the drawers and cupboards are in the way. That's really frustrating, because what I should be doing is working on the issue of carbon sequestration in the southern oceans.”*

This implies that valued outcomes must move beyond fragmented functional and independence measures which attend to capacities such as “mobility” as a discrete function and encompass emerging conceptualisations of life domains [19, 27].

The evidence presented supports the premise that combining interventions into tailored AT solutions is effective as this is in fact how they are used in life. Also, that a lack of joined up service provision exists in Victoria, Australia, along with significant underresourcing of government equipment provision schemes.

This study confirms other recent evidence in identifying that optimal mobility depends upon adequate provision of a range of enablers at both person and environment level [32, 33]. A multilevel or systems approach is required to address these levels [13, 33]. Those practitioners working with people with disabilities to enable participation must also attend to the world in which the person and their mobility device engage. Beyond the garden gate, people operate within broader environments. Facilitators such as continuous paths of travel within the community, accessible public transport networks, welcoming buildings with operable doors and lifts, accessible counters, and educated and friendly staff are essential ingredients for full participation beyond the home. 

Who is responsible for the broader community environs? Duty holders in this respect may include local government authorities, government departments dealing with such infrastructure as transport, or individual businesses. While complaints to antidiscrimination bodies such as equal opportunity or human rights commissions can help provide redress for individuals unlawfully denied access to infrastructure or services, proactive measures by these stakeholders are necessary to address the systemic barriers which create inaccessible environments. Rehabilitation practitioners are key stakeholders in the business of enablers for mobility. Occupational therapists, physiotherapists, and other prescribers of AT devices and environmental adaptations to the home rarely advise more widely regarding the built environment. Arguably, practitioners must take on roles beyond individual advocate, towards the more political practice of systemic advocacy [34].

Several other developments may hold answers for improving other facets of inclusive community. The universal design and inclusive design movements [33, 35] offer to both minimize the costs of retrofitting for access through better planning and to destigmatise many enabling elements which can benefit entire communities [36]. From an economic perspective, several authors are beginning to investigate the costs and impacts of nonsocial design [37–39]. This approach potentially allocates costs of access and inclusion out to all community dwellers likely to benefit, so for example parents with prams and retirees share in the benefits of accessible transport and inclusive communities alongside people living with disabilities. 

### 4.1. Limitations of the Study

This study provides a snapshot of enabler-use (broadly defined) and unmet need for a cohort of Victorians with disabilities. It is likely that people with disabilities living outside Victoria or indeed Australia would have similar experiences, although the contextual factors such as government funding or legislative support for community access are likely to differ in other jurisdictions. Efforts were made to ensure the survey tool was universally accessible; however the sample consists largely of adults on substantially low incomes and with low employment rates. Those whose cognition and language skills were insufficient for independent reporting were excluded. A significant issue with the survey design was missing demographic data, and in future studies it would be advisable to place the demographic section at the commencement of the survey, while monitoring impacts on completion particularly for severely impaired participants. 

## 5. Conclusion

Individuals living with impairment face barriers on many levels. In researching community mobility, a valuable perspective is gained through examining barriers and facilitators at both person and environment level. On an individual level, access to tailored enablers is not guaranteed by government equipment schemes despite theoretic commitment to frameworks such as the ICF and the CRPD. From a societal standpoint, barriers remain present within environments and both political and community action be required to transform these environmental barriers into facilitators. If rehabilitation practitioners are committed to optimising community mobility, then the locus of attention must expand to encompass both the individual as well as broader elements of the environment.

## Figures and Tables

**Table 1:** Description of Research methods.

Research question	Research methods	Administration and tools
Oversight and triangulation	a. Stakeholder reference committee	Face to face meetings (4)
	Ethics Approval	Deakin University
	Survey pilot	*n*-9

Survey sample (*n*-100)		

Demographics of respondents	Demographic questions	Survey section 3: 12 questions
How are AT, EI and PC used in relation to each other and what else is enabling?	Use of supports and improvement over life domains	Survey section 1: 80 questions
How does the presence or absence of enablers effect life for people with disabilities?		
Costs aside, what would improve life?	Health-related Quality of Life measure	Survey section 2: AQoL 6D (20 questions)

**Table 2:** Mobility device useage and unmet need (N-100).

AT used for mobility categorised by ISO 9999 Chapters	Useage	Unmet need
12 03 & 12 06 Assistive products for walking		
(i) Walking sticks	11	
(ii) Walking frames	17	1
(iii) Crutches	2	
12 12 Car adaptations		
(i) Vehicle modifications	36	6
(ii) Vehicle transfer aids	2	1
(iii) Vehicle seating & restraints	6	
12 18 Cycles		1
12 22 Human-driven wheelchairs	48	11
12 22 Powered wheelchairs (including scooters)	50	7
12 24 Wheelchair accessories (includes conversion kits, trays, postural supports)	14	9
24 36 Assistive products for carrying and transporting		
(i) Lifters, carriers & trailers	13	4
(ii) Scooter/wheelchair hoists	3	3

**Table 3:** Unmet need for accessible community environs.

ICF Chapter; Issues and Examples	Supporting quotes (verbatim)
ICF Chapter 2 Public buildings	“When buildings are renovated or first built of they should have ramps, easy opening doors, access to upper floors and counters that are accessible to people in wheelchairs”
Issue: “Universal design in all public buildings, and private buildings designed for the public for example, hotels, restaurants”	“easy access to buildings would save huge amounts of time and stress”
Examples:	
Stepless entry;	(I want to) “Have a choice about what cafes and shops I go to. Freedom not to plan my every move.”
Easy open doors;	
Accessible toilets;	“If more workplaces were wheelchair friendly then maybe people with disabilities would be more easily included in work!”
Appropriate height reception/sales desks at shops and other venues;	
Seating;	“a bad access example is when i go to vote. I have trouble getting into the building and I need a lot of help as access is through a very steep temporary ramp… i need to go with a carer”
Accessible swimming pools/gyms	

Instances of unmet need reported: 48	
Percentage of unmet need (36%)	

ICF Ch 2 &5 Public transport	“A change to disabled parking would be most beneficial—as I cannot park in ordinary parking bays, I have to wait sometimes up to two hours for a disabled parking bay”
Issue: “Travel more freely” Examples: More low floor buses Accessible tram stops	[I need] “plenty of places to sit and rest, public transport stops closer together” “My church is in Melbourne and no trams there yet—got the stops but no accessible trams on that line!” (I need) “A nearby low floor bus. Closest bus is not accessible. 2.5 Km to nearest accessible bus. Many places unable to go to directly by bus as no low floor bus”
Large print and talking timetables	

Instances of unmet need reported: 31	
Percentage of unmet need 23%	

ICF Chapter 2 Public Space	“CBD parking in Melbourne not one on steet (*sic*) park meets the requirements in regards to the width, as my car is fitted with a wheel chair hoist on the roof and i find it difficult to find a park where I'm not lowering my chair into oncoming traffic.”
Issue: “Get out and about… get to things”	“Accessible milk bar nearby—the three I could use (in different directions) all have steps. Improved footpath crossings at intersections”
Examples:	
Continuous paths of travel	(I need) “a cut in path in my nature strip near my front door as the nearest cut in the gutter is up the road which when getting a maxitaxi I get rather wet, council will not let me do it even though I was willing to pay”
Footpaths	
Kerb access	
Tactile street signage	“Ongoing need for improvements in footpaths and crossovers everywhere.”
Street crossings	
Accessible parking	

Instances of unmet need reported: 32	
Percentage of unmet need: 24%	

ICF Ch 3,4,5 Public information and support Issues: Accessible information Examples: Accessible websites Venue access info Helpful trained staff	“An impossible change—peoples attitudes, just because I am in a chair I am not stupid!!!!” “Accessible hotels/holiday venues: this area needs a huge change to accommodate people with disabilities. Accommodation venues state that they are accessible but they are not or do not meet the Standards. In my case, I will not now go to a venue unless I see photographs of the toilet and shower to ascertain if I will be able to manage when I get there”
